# MtlR negatively regulates mannitol utilization by *Vibrio cholerae*

**DOI:** 10.1099/mic.0.000559

**Published:** 2017-10-27

**Authors:** Tanner Byer, Jessica Wang, Mark G. Zhang, Naomi Vather, Anna Blachman, Bryan Visser, Jane M. Liu

**Affiliations:** Department of Chemistry, Pomona College, Claremont, CA 91711, USA

**Keywords:** mannitol, *Vibrio cholerae*, phosphotransferase system

## Abstract

The phosphoenopyruvate:carbohydrate phosphotransferase system (PTS) enables *Vibrio cholerae* – and other bacteria – to recognize and transport exogenous carbon sources for energy, including the six-carbon sugar alcohol, mannitol. The mannitol-specific PTS transporter is encoded by *mtlA* and its expression is expected to be regulated by the putative repressor encoded by the *mtlR* gene. Here, we show that *mtlR* overexpression inhibits *V. cholerae* growth in medium supplied with mannitol as the sole carbon source and represses MtlA-mediated biofilm formation. We demonstrate that when *V. cholerae* is grown in non-mannitol medium, knocking out *mtlR* leads to both increased MtlA protein and *mtlA* mRNA levels, with these increases being especially pronounced in non-glucose sugars. We propose that in non-mannitol, non-glucose growth conditions, MtlR is a major regulator of *mtlA* transcription. Surprisingly, with regard to *mtlR* expression, transcript and protein levels are highest in mannitol medium, conditions where *mtlA* expression should not be repressed. We further show that MtlR levels increase during growth of the bacteria and linger in cells switched from mannitol to non-mannitol medium. Our data suggests an expression paradigm for *mtlA* where MtlR acts as a transcriptional repressor responsible for calibrating MtlA levels during environmental transitions.

## Introduction

As a facultative pathogen, *Vibrio cholerae* thrives in both marine ecosystems and the gastrointestinal tract of a human host [1–3]. *V. cholerae* colonization of these disparate climates requires molecular mechanisms to reconcile fluctuations in temperature, pH and carbon source availability [4–8]. One method of adaptation is the recognition and utilization of various carbohydrates for energy, which necessitates the phosphoenolpyruvate (PEP):phosphotransferase system (PTS) [9–12]. Highly conserved across bacteria, the PTS responds to environment-specific stimuli through a phosphorylation cascade [13, 14]. Enzyme I (EI) first transfers a phosphoryl group from PEP to histidine protein (HPr). HPr proceeds to phosphorylate one of several EIIA proteins. Whereas cytosolic EI and HPr are common, core PTS components, sugar transport is facilitated by carbohydrate-specific EII proteins [13]. In *V. cholerae*, nine EIIA proteins have been identified and are each specific to different carbohydrates [10, 15]. The EIIA protein assembles, with EIIB and EIIC subunits, into a larger permease complex (EII) that catalyses the final step of the PTS: concomitant transport and phosphorylation of the corresponding, extracellular carbon source [13].

For most bacteria, including *V. cholerae*, transport of exogenous d-mannitol requires the PTS. d-mannitol is a six-carbon sugar alcohol produced by marine algae and is the most abundant hexitol found in nature [16–19]. The mannitol-specific PTS transporter (EII^Mtl^) is encoded by genes at the mannitol (*mtl*) locus of both Gram-negative and Gram-positive bacteria. This locus also contains *mtlD*, encoding a dehydrogenase responsible for oxidizing newly acquired and phosphorylated mannitol (mannitol-1-phophate) into fructose-6-phosphate, the successive metabolic intermediate [20]. In the case of *Escherichia coli* and other proteobacteria, the *mtl* locus also encodes *mtlR*, expressing a regulator of the *mtl* genes [21]. *mtlR* is also expressed from the *mtl* locus of firmicutes, but in the case of *Bacillus subtilius*, the gene encoding the regulator is located 15 kb downstream from the rest of the *mtl* genes [22].

The *mtl* genes of *V. cholerae* span 3.9 kb and are organized as *mtlADR. mtlA* encodes the EII^Mtl^ PTS transporter, which shares high sequence homology to the extensively studied *E. coli* MtlA [23]. In both species, the EIIC^Mtl^, EIIB^Mtl^ and EIIA^Mtl^ subunits are all encoded by the *mtlA* gene and are fused to each other, in that order [20, 23]. MtlA activity is required for mannitol metabolism in *V. cholerae*; bacteria lacking functional MtlA are unable to survive in media supplemented with mannitol as the sole carbon source [23, 24]. The persistence of *V. cholerae* in marine ecosystems is also bolstered by MtlA activity; the EIIB subunit of MtlA is capable of inducing biofilm formation, potentially providing the bacteria a fitness advantage in its aquatic reservoir [3, 25].

Regulation of *mtlA* in *V. cholerae* is complex and involves the 3′,5′-cyclic adenosine monophosphate (cAMP) receptor protein (CRP). This regulator is activated by cAMP to directly bind five distinct sites found in the *mtlA* promoter and is necessary for activation of *mtlA* [26]. Recent studies further demonstrate that *mtlA* expression in *V. cholerae* is negatively regulated by MtlS, a *cis*-acting small regulatory RNA (sRNA) [27, 28]. One of the least characterized regulators of *mtlA* in *V. cholerae*, however, is MtlR.

Based on genomic annotation, the *V. cholerae mtlR* gene codes for a 195-amino acid protein (22 kD) that shares sequence homology (63 %) to the *E. coli* homologue [23, 29]. MtlR activity was first studied in the *E. coli* system, where the protein demonstrated the ability to repress the uptake of exogenous mannitol [29]. In line with this, knocking down *mtlR* expression in *V. cholerae* allows the bacteria to achieve exponential growth more quickly when transitioned from rich medium to a mannitol-only medium; this activity appears to be independent of MtlS [28, 30]. It has been hypothesized that MtlR-mediated repression involves the protein directly binding to DNA operators in the *mtlA* promoter, but interactions between MtlR and DNA have yet to be observed [29, 31]. The solved structure of MtlR from *Vibrio parahemeolyticus*, furthermore, reveals an absence of established DNA-binding motifs and electrostatic properties unlikely to accommodate direct DNA binding [31]. Thus, the mechanistic details explaining how MtlR affects mannitol metabolism in proteobacteria remain largely unknown.

For the present study, we sought to further investigate the activity and regulation of MtlR to better understand the collective modulation of *mtlA* expression in *V. cholerae*. We investigated the effect of MtlR activity on the physiology of *V. cholerae* exposed to exogenous mannitol. Additionally, we considered the expression profile of *mtlR* in the presence and absence of mannitol. Our results suggest that in certain environments, MtlR represses the transcription of *mtlA* and, therefore, the ability of *V. cholerae* to respond to extracellular mannitol. We also establish that the expression of *mtlR* is unexpectedly increased in the presence of mannitol, where it accumulates throughout growth. We suggest a model in which MtlR may serve to fine-tune MtlA levels when *V. cholerae* transition between areas where mannitol concentrations fluctuate.

## Methods

### Strain and plasmid construction

All strains and plasmids used in this study are listed in Table S1 (available in the online version of this article). The *V. cholerae* strains used in this work were El Tor strain N16961 ∆*tcpA* and derivatives (Table S1). The *tcpA* mutant is highly attenuated for virulence and was used for safety purposes [32]. Throughout this text, ‘wild-type’ refers to the N16961 ∆*tcpA* strain. Plasmids with *oriR6K* were propagated in *E. coli* DH5αλpir; all other plasmids were propagated in *E. coli* DH5α or TOP10 strains. Oligonucleotide primers and labelled probes used in this study are listed in Table S2 and were acquired from Integrated DNA Technologies.

Plasmids for generating chromosomal mutations in *V. cholerae* were constructed in the allelic exchange vector pCVD442 [33]. The *mtlR–HA* mutant was constructed by combining, using Gibson Assembly [34], two 500 bp gBlock fragments (Integrated DNA Technologies) that introduced the sequence encoding the HA tag at the C-terminus of *mtlR*. The resulting DNA was PCR-amplified with F1 and R2 primers and cloned into the multiple cloning site of pCVD442 using the SphI and SacI restriction sites. All pCVD442-derived constructs were conjugated into *V. cholerae* N16961 ∆*tcpA* from *E. coli* SM10λpir as described previously [35]. After one passage in Luria–Bertani (LB) broth with streptomycin, sucrose-resistant colonies were selected and subsequently screened for the desired mutation by PCR and sequencing.

Plasmids harbouring *mtlR* under the control of the P_trc_ promoter were constructed by PCR amplifying *mtlR* from *V. cholerae* N16961 ∆*tcpA* using oligonucleotides containing SacI and XbaI restriction sites. PCR products and vector pTrc99A were digested with SacI and XbaI restriction endonucleases. Digestion products were ligated using T4 DNA ligase to produce plasmids containing P_trc_-*mtlR* alleles.

### Bacterial growth

All strains were grown under shaking conditions at 250 r.p.m. at 37 °C in LB medium or M9 minimal medium supplemented with 0.1 % trace metals (5 % MgSO_4_, 0.5 % MnCl_2_•4H_2_O, 0.5 % FeCl_3_, 0.4 % nitriloacetic acid) and 0.4 % carbon source. Throughout the text, ‘glucose medium’, ‘mannitol medium’, etc., refer to M9 minimal medium supplemented with trace metals and 0.4 % of the respective carbon source. When necessary, cultures were supplemented with 1 mM IPTG to induce the expression of genes inserted into the pTrc99A plasmid under the control of the P_trc_ promoter. Antibiotics were used at the following concentrations: streptomycin, 100 µg ml^−1^; carbenicillin, 50–100 µg ml^−1^.

### Growth curves

Growth of each strain was determined by measuring OD_600_ using a Bio-Tek microplate reader. Colonies were collected from LB agar plates and grown in LB medium for 2–3 h at 37 °C, 250 r.p.m. Cells were then pelleted and washed with 1× M9 salts, and the OD_600_ was adjusted to 0.01 (~10^7^ c.f.u. ml^−1^) in 200 µl of supplemented M9 medium with 0.4 % of the appropriate carbon source. Bacteria were grown with orbital shaking at 37 °C for 12–18 h in the microplate reader at 37 °C. All growth experiments were performed in at least triplicate.

### Biofilm assay

Biofilm formation by *V. cholerae* was quantified as previously described, with minor modifications [25]. Strains were first grown overnight on LB agar plates at 37 °C; colonies were then used to inoculate 2 ml LB cultures, which were grown at 37 °C, 250 r.p.m. After 6–8 h, the cultures were used to inoculate 300 µl of LB medium in borosilicate tubes. After incubation for 17 to 24 h at 22 °C, each planktonic cell suspension was collected and its OD_655_ was measured using a Bio-Tek microplate reader. To quantify the surface-attached cells, the tubes were rinsed with PBS, followed by the addition of 300 µl of PBS and a small volume of 1 mm diameter glass beads (Biospec,). Through vortexing, the attached cells were dispersed and the OD_655_ of the resulting cell suspension was measured. The sum of the OD_655_ values measured for planktonic and surface-associated cell suspensions are reported as the total growth of the cells. Statistical analysis was performed using GraphPad Prism 6 software.

### RNA analysis

*V. cholerae* were cultured to an OD_600_ ~0.3 and total RNA was extracted using the DirectZol RNA MiniPrep Kit (Zymo) following the manufacturer’s suggested protocol. DNA was further removed from all samples using the TURBO DNA-free kit (Thermo Fisher Scientific), according to the manufacturer’s instructions. To analyse the nature of the mRNA transcripts from the *mtl* locus, reverse transcription-PCR (RT-PCR) was used. RT of total RNA (100 ng) was performed with reverse primers (2 pmol) specific to *mtlADR* using Superscript III (Thermo Fisher Scientific) according to the manufacturer’s instructions. The cDNA generated or N16961 genomic DNA was used as a template for PCR with reverse and forward primers specific to monocistronic transcripts or *mtlAD*, *mtlDR* or *mtlADR* polycistronic transcripts. PCR reactions were performed with an initial denaturation step of 5 min at 95 °C, followed by 26 cycles of 30 s at 95 °C, 30 s at 55 °C and 4 m at 72 °C. Control reactions of RNA samples not treated with reverse transcriptase were performed for each run; no product band was observed in any of the no-RT controls.

qRT-PCR was used to analyse relative expression levels using gene-specific primers, a Stratagene MX3005P System, and the Brilliant II SYBR Green qRT-PCR Master Mix Kit (Agilent). The reactions were set up in 96-well optical reaction plates and contained 1× Brilliant SYBR Green qPCR Master Mix, 30 nM ROX reference dye, each primer at 100 nM, 100 ng RNA and 1 µl RT/RNase block enzyme mixture in a 25 µl reaction. The following conditions were used for cDNA synthesis and PCR: 30 m at 50 °C, 10 m at 95 °C, and 40 cycles of 30 s at 95 °C and 1 m at 60 °C (Agilent). MxPro QPCR software (v. 4.10) was used to determine the Ct for each reaction. Relative RNA concentrations were calculated from the Ct values by comparison to standard curves; the relative transcript levels were normalized to an endogenous control (4.5S RNA). No signals were detected in no-template controls and no-RT controls. Statistical analysis was performed using GraphPad Prism 6 software.

### Western blot analysis

Bacteria were grown to an OD_600_ ~0.3 and collected (8000 ***g***, 5 min). For MtlA analysis, lysates were prepared by mixing ~10^7^ cells with SDS-loading buffer and heating at 95 °C for 10 min. For MtlR analysis, cell pellets from 5 to 50 ml cultures were lysed with B-PER Extraction Reagent (Thermo Fisher Scientific) in the presence of DNAse I, following the suggested protocol of the manufacturer; the lysate was then mixed with SDS-loading buffer. The proteins were resolved on 4–20 % TRIS gels (Bio-Rad) and transferred to nitrocellulose membranes. The membranes were treated with polyclonal anti-FLAG (abCam), polyclonal anti-HA (abCam), monoclonal anti-RpoB (abCam), or monoclonal anti-RNAPα (BioLegend) antibodies. Antibodies were detected using secondary antibodies with IR680 and IR800 dyes attached (Licor). As a membrane protein, MtlA has a tendency to oligomerize, even under the denaturing conditions of SDS-PAGE. Signals were visualized and band densities were measured using an Odyssey imager (Licor).

## Results

### MtlR represses the ability of *V. cholerae* to use and respond to extracellular mannitol

**Fig. 1.**
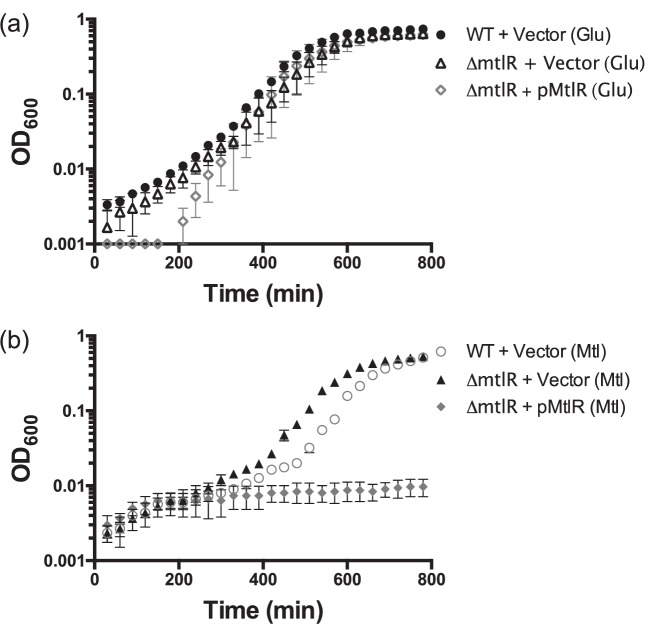
Overexpression of *mtlR* negatively impacts growth of *V. cholerae* on mannitol medium. (a, b) Growth curves of the indicated strains in M9 medium supplemented with the indicated carbon source. Bacteria were first grown to mid-exponential phase in LB medium. Cultures were washed and used to inoculate 200 µl M9 medium with 1 mM IPTG and either glucose (Glu, 0.4 %) or mannitol (Mtl, 0.4 %). Strains were grown at 37 °C with orbital shaking in a 96-well plate. The error bars represent the standard deviation of three biological replicates.

Strains lacking *mtlR* have a slightly shorter lag phase, compared to the wild-type, when transitioned from LB to mannitol medium [28], conditions that allow one to assess the effect that MtlR has on cells adapting to new conditions that may include mannitol as the primary carbon source. We attempted to complement this phenotype by introducing an *mtlR* overexpression plasmid into the knockout strain (Fig. S1). This strain had difficulty adapting to mannitol medium (Fig. 1a, b). We hypothesize that the overexpression of *mtlR* in mannitol prevents the bacteria from producing enough MtlA to take up adequate levels of mannitol to meet their metabolic needs. The effects of overexpressing or deleting *mtlR* on cell growth appear to be specific to mannitol and not a general effect on carbon uptake or metabolism as all strains grew similarly in glucose medium, although an extended lag phase was observed in the wild-type strain harbouring pMtlR.

**Fig. 2.**
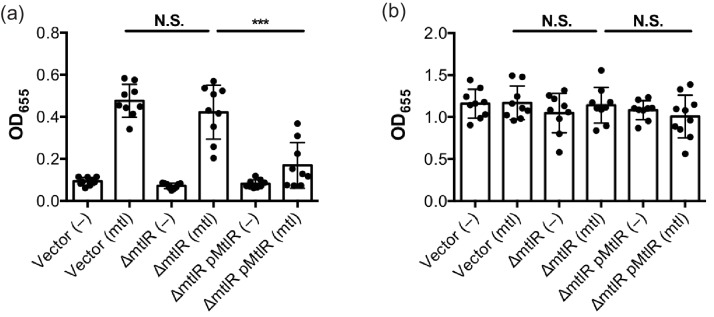
MtlR negatively affects mannitol-induced biofilm formation. Quantification of (a) surface-attached cells and (b) total cell growth of the indicated strains. Wild-type *V. cholerae* or an isogenic ∆*mtlR* strain harbouring either an empty vector control or pMtlR. Cells were cultured in LB medium supplemented with mannitol (mtl, 0.5 %) or an equivalent volume of water (–). IPTG (1 mM) was also added to induce expression from the pTrc99A vector. Bar graphs represent the means and sds from multiple independent samples. ***P<0.001; ns, not significant, by unpaired two-tailed *t*-test.

Expression of the B subunit from the EII^mtl^ protein has been shown to activate biofilm formation in *V. cholerae*. Consistent with prior studies, the *mtlR* knockout behaves similarly to the wild-type in that both strains increase biofilm formation in response to mannitol (Fig. 2a, b) [25]. Overexpression of *mtlR* in the knockout strain, however, repressed the ability of the bacteria to induce biofilm formation when mannitol was added to the growth medium, without affecting overall growth (Fig. 2a, b). In summary, both the growth curve and biofilm assay results are consistent with MtlR acting as a negative regulator of *mtlA* in *V. cholerae*.

### MtlR downregulates *mtlA* expression at the transcriptional level

**Fig. 3.**
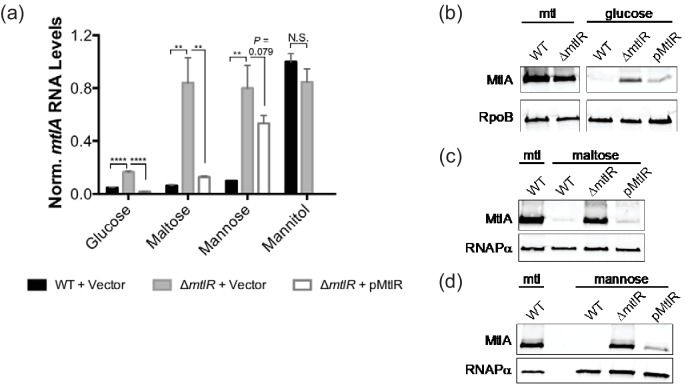
MtlR represses *mtlA* expression. Wild-type *V. cholerae* harbouring an empty vector (WT), an isogenic strain lacking *mtlR* (∆*mtlR*) and a ∆*mtlR* strain harbouring pMtlR (pMtlR) were cultured in M9 minimal medium with glucose, mannitol (mtl), maltose or mannose (0.4 % w/v) at 37 °C to an OD_600_ of 0.3; IPTG was not added to the cell cultures. (a) qRT-PCR was used to measure *mtlA* mRNA levels, which were first normalized to 4.5S RNA levels, and then to *mtlA* levels in the wild-type strain grown in mannitol medium, as described in Methods. Analysis of *mtlA* mRNA in mannitol medium focused on only the wild-type and knockout strains. Bar graphs represent the means and sds from three biological replicates. ** *P*<0.01; ****P<0.0001; ns, not significant, by unpaired two-tailed *t*-test. (b–d) MtlA-FLAG protein levels were analysed by Western blot. Cell lysates from an equal number of cells were analysed using anti-FLAG, anti-RpoB and anti-RNAPα antibodies. RpoB and RNAPα served as a loading control. For (b), data is from different regions of the same blot. All data are representative of three independent trials.

We previously reported conflicting results from qRT-PCR and Northern blot analyses on *mtlA* mRNA levels in glucose versus mannitol medium [24, 28]. We designed a new probe for *mtlA* mRNA (Fig. S2) and Northern blotting analysis using this probe entirely agrees with qRT-PCR: *mtlA* mRNA levels are up-regulated in mannitol medium, compared to glucose medium (Figs 3a and S3). We reasoned that the probes that we used in our previous report were binding to a non-specific target and that the new probes we used here accurately report relative *mtlA* mRNA levels in *V. cholerae*.

To investigate the role of MtlR in *mtlA* transcription, we repeated the qRT-PCR analysis with an isogenic strain lacking *mtlR*. In mannitol medium, knockout of *mtlR* did not lead to higher levels of *mtlA* transcript or MtlA protein, confirming our expectation that MtlR has no major activity in mannitol growth conditions (Fig. 3a, b). These results are consistent with our growth curve and biofilm data. We therefore focused our efforts on assessing the role of MtlR in non-mannitol growth conditions.

In glucose (conditions where *mtlA* expression is normally repressed), there was a small but significant increase in *mtlA* transcript levels when *mtlR* was knocked out (Fig. 3a). We observed a similar pattern in MtlA protein levels – knocking out *mtlR* significantly increased MtlA protein levels compared to wild-type, albeit only to 30 % of the amount found in *V. cholerae* grown in mannitol medium (Fig. 3b). These results suggest that MtlR plays a minor role in regulating *mtlA* at the transcriptional level when *V. cholerae* are grown in glucose medium and are consistent with our above analysis that suggested MtlR as a repressor of *mtlA*. We were able to partially complement the *mtlR* mutation by introducing *mtlR* on a plasmid in the mutant strain, indicating that it is the gene product of *mtlR* that directly impacts MtlA synthesis (Fig. 3a, b). In these complementation experiments, and the ones below, in order to obtain more physiologically relevant levels of MtlR we did not add IPTG to the growth medium (Fig. S1).

In addition to its role in transporting glucose into the cell, EIIA^Glc^ indirectly regulates CRP [13], a known transcriptional activator of *mtlA* [26]. To avoid the complication of perturbing a major transcription factor known to activate *mtlA*, we chose to also test the effects of *mtlR* on *mtlA* transcription in non-glucose, non-mannitol growth conditions. When the ∆*mtlR* strain was grown in maltose medium (a non-PTS sugar) and mannose medium (a PTS-sugar), we observed that *mtlA* transcript levels were highly elevated compared to the wild-type controls (Fig. 3a). Ectopic expression of *mtlR* was able to partially complement this phenotype (Fig. 3a). Western blot analysis of these same strains grown in maltose or mannose medium further supports a role in which MtlR is a major regulator of *mtlA* expression in non-mannitol, non-glucose medium (Fig. 3c, d).

### Expression of *mtlR* is induced in mannitol and accumulates throughout growth

**Fig. 4.**
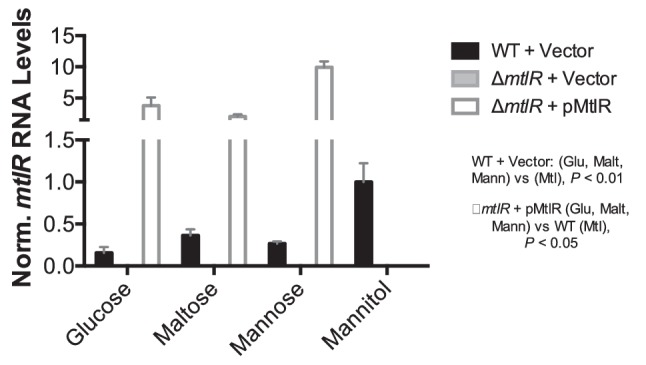
*mtlR* mRNA levels increase in mannitol medium. The same samples as in Fig. 3 were used to measure *mtlR* mRNA levels via qRT-PCR. *mtlR* RNA levels were normalized to 4.5S RNA levels, and then to *mtlR* levels in the wild-type strain grown in mannitol medium, as described in Methods. Analysis of *mtlR* mRNA in mannitol medium focused on only the wild-type and knockout strains. Bar graphs represent the means and sds from three biological replicates. *mtlR* mRNA levels were undetectable, as expected, in the mutant strain. Statistical significance was determined by unpaired two-tailed *t*-test. All data are representative of three independent trials.

**Fig. 5.**
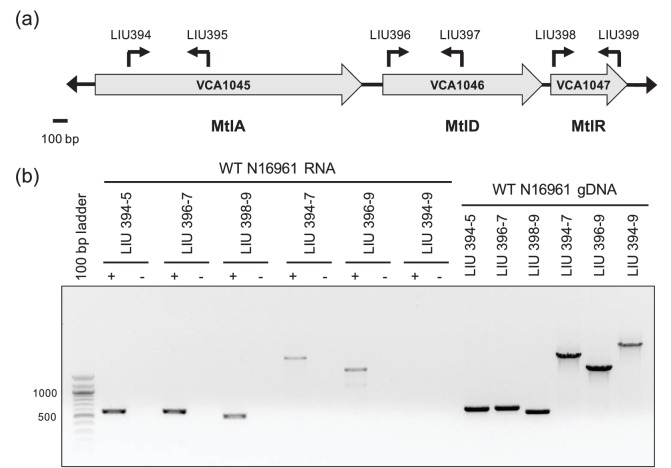
*mtlAD* and *mtlDR* are co-transcribed. (a) Schematic representation of the 3.9 kb *mtl* locus in *V. cholerae* including the *mtlA*, *mltD* and *mtlR* genes, their transcriptional directionality, and the location and names of primers used to determine transcriptional organization. (b) RT-PCR products from total RNA collected from *V. cholerae* grown in mannitol medium were analysed by gel electrophoresis on an agarose gel. + and − correspond to reactions run in the presence and absence of reverse transcriptase, respectively. PCR was also performed on *V. cholerae* genomic DNA (gDNA) to provide positive controls. Data are representative of three independent trials.

We expected, given the activity of MtlR, that it would be most highly expressed in non-mannitol medium. Instead, we saw that *mtlR* mRNA levels increase in mannitol (Figs 4 and S4). This may be a result of increased expression of the entire *mtl* locus during exposure to mannitol. To test this theory, we used RT-PCR to determine the transcriptional organization of the *mtl* locus when the bacteria were grown in mannitol medium. RT-PCR primers were designed to amplify coding regions spanning two or more *mtl* genes to probe for the presence of polycistronic transcripts (Fig. 5a). Affirming that all *mtl* genes were expressed under these growth conditions, the expected RT-PCR products for *mtlA* (576 bp), *mtD* (585 bp) and *mtlR* (515 bp) were detected (Figs 5b and S5). RT-PCR products for *mtlAD* (2560 bp) and *mtlDR* (1748 bp) products were also observed, but no product from an *mtlADR* transcript was obtained, despite multiple attempts (Fig. 5b). These results suggest a transcriptional paradigm in which the *mtlD* gene can be co-expressed with *mtlA* and *mtlR* individually, but the three gene units do not necessarily form a single polycistronic transcript. The existence of multiple promoters within a single operon is a genetic structure that has been previously reported in *V. cholerae* [36, 37]. Alternatively, the *mtlADR* transcript may be rapidly processed into smaller transcripts. The primer pair we use for the *mtlADR* transcript also appears to be less efficient, which may have negatively impacted our ability to observe the *mtlADR* transcript.

**Fig. 6.**
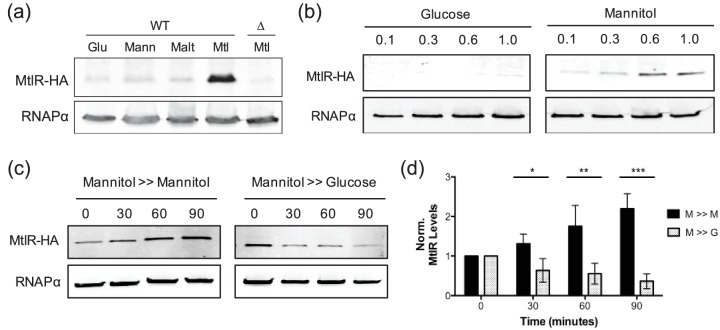
MtlR protein levels are higher in mannitol medium compared to glucose medium. (a) MtlR-HA protein levels from bacteria (wild-type, WT; isogenic ∆*mtlR*, ∆) cultured to an OD_600_ of 0.3 at 37 °C in M9 minimal medium with the indicated carbon source (0.4 %): glucose (Glu), mannose (Mann), maltose (Malt), mannitol (Mtl). (b) Wild-type *V. cholerae* cells cultured at 37 °C in M9 minimal medium with glucose or mannitol (0.4 %) were harvested at the indicated OD_600_. (c) Wild-type *V. cholerae* were cultured at 37 °C in M9 minimal medium with 0.4 % mannitol. At an OD_600_ of 0.3, cells were collected, re-suspended in an equal volume of M9 minimal media with 0.4 % mannitol or glucose and harvested at 0, 30, 60 and 90 min relative to the medium switch. (d) Quantification of MtlR-HA from Western blot analyses, as in (c), normalizing to RNAPα levels and then to MtlR-HA levels at time point 0. Shown are the means and sds from three independent experiments. *P<0.05; **P<0.01; ***P<0.001 by unpaired two-tailed *t*-test. For Western blots, whole cell lysates from an equal number of cells were analysed by Western blot with anti-HA and anti-RNAPα antibodies. RNAPα is acting as the loading control. All blots are representative of at least three independent trials.

In order to assess MtlR protein levels, we first determined that the N-terminus of *mtlR* was previously mis-annotated, and that the correct annotation for *mtlR* encodes a 177-amino acid protein (Figs S6 and S7). Using a strain harbouring an HA-tagged *mtlR*, we investigated the profile of MtlR in cells cultured to mid-exponential phase in various growth media. Consistent with the *mtlR* mRNA analysis, we observed the surprising result that MtlR protein levels were markedly elevated in mannitol growth medium; in non-mannitol conditions, the level of MtlR protein was determined to be ~5 % of that found in mannitol medium, as determined by quantification of MtlR band densities (Fig. 6a).

Our initial analysis of *mtlR* expression was based on analysing bacteria grown to mid-log phase (OD_600_ ~0.3). We considered that at different growth phases, the bacteria might exhibit a more expected expression profile, with MtlR levels higher in non-mannitol medium compared to mannitol medium. Thus, cells were grown to varying optical densities in glucose and mannitol medium and subsequently analysed by Western blot (Fig. 6b). Although these data suggest that cellular MtlR levels are highest at elevated optical densities regardless of the carbon source, the levels of MtlR appear to be higher in cells grown in mannitol medium versus glucose medium at any point during growth.

Given the unexpected expression pattern of *mtlR*, we considered that the bacteria may maintain high levels of the repressor during transitions from mannitol to non-mannitol growth conditions to assist with adaptation. To test this theory, we cultured cells in mannitol medium to an OD_600_ of 0.3 before switching the growth conditions to either fresh glucose or mannitol medium. *V. cholerae* cells were harvested at time points subsequent to the media switch and MtlR levels were analysed. Upon moving bacteria from mannitol to glucose medium, we observe a marked decrease to approximately 60 % of initial MtlR levels 30 min after the media switch (Fig. 6c, d). MtlR levels linger around 50 % of initial measurements 60 and even 90 min after the media switch to glucose growth medium (Fig. 6c, d). Similar results were observed when moving the bacteria from mannitol into maltose or mannose medium (Fig. S8). In terms of MtlA, we generally observe a consistent decrease in transporter levels after moving *V. cholerae* from mannitol to glucose medium (Fig. S8) [28]. That MtlR protein levels remain appreciable up to 1.5 h subsequent to a media switch out of mannitol suggests that, in *V. cholerae,* MtlR assists with adaptation to new, non-mannitol media by precluding *mtlA* expression during the early stages of transition.

## Discussion

Regulation of *mtlA* expression in *V. cholerae* is a process involving a range of different cellular machinery, including the global regulator CRP [26] and the *cis*-acting small regulatory RNA, MtlS [27, 28]. Taken together, the established framework for *mtlA* regulation includes both transcriptional and post-transcriptional mechanisms. Here, we characterize a third regulator of *mtlA* expression: MtlR, a transcriptional regulator of *mtlA* that plays a role in regulating mannitol transporter levels in non-mannitol growth conditions. Our results are further supported by a study from Wang *et al*., suggesting that MtlR acts as a transcriptional regulator of *mtlA* expression in both toxigenic and non-toxigenic El Tor strains of *V. cholerae* [30]. We note, however, that the role of MtlR does not appear to be equal in all non-mannitol media. In glucose, for example, MtlR appears to play a minor role in regulating *mtlA* transcription (Fig. 3a). Given the known role of CRP in activating *mtlA* expression, it could be that in glucose growth conditions, *mtlA* transcription is largely governed by cAMP levels – low adenylyl cyclase activity results in low *mtlA* mRNA levels. However, in the case of mannose and maltose, MtlR appears to be the major transcriptional repressor of *mtlA* (Fig. 3a). Indeed, in these non-glucose, non-mannitol conditions, MtlR may counteract elevated cAMP levels to maintain low *mtlA* expression when the transporter is not needed.

Our data also suggest MtlR represses *mtlA* expression with considerable efficacy. MtlR levels in *V. cholerae* grown to mid-exponential phase in non-mannitol conditions are almost imperceptible in our traditional Western blot assay, but necessary for full repression of *mtlA* expression (Figs 3 and 6). This being said, MtlR alone is insufficient for full repression of *mtlA,* as evident by our observations that maximal levels of MtlA are not observed in ∆*mtlR* bacteria grown under non-mannitol conditions (Fig. 3). It is likely that MtlR acts additively alongside MtlS, an sRNA that is similarly expressed under non-mannitol conditions and also represses *mtlA*. MtlR- and MtlS-mediated repression of *mtlA* appear to be independent of each other, moreover, as MtlR exhibits no observable influence on MtlS synthesis [28].

We also noted that our complementation strain was not able to bring *mtlA* expression back to wild-type levels. This could be because the complementation assays were done in the absence of IPTG, which does allow for ectopic *mtlR* expression, but at low levels (Fig. S1). Alternatively, there may be some *cis*-effects with regard to MtlR’s regulation of *mtlA*. For example, chromosomal *mtlR* may allow for MtlR to be synthesized and localized to specific regions of the cell that would allow for maximal activity. The *mtlR* locus may also express additional transcripts that affect *mtlA* expression, which were not appropriately expressed from the pMtlR construct.

As a repressor of *mtlA*, excess MtlR protein can impair *V. cholerae* growth in mannitol medium. Accordingly, we observed that ectopic expression of *mtlR*, even in the absence of IPTG, can impede growth of the bacteria when initially switched from LB to mannitol medium (data not shown). We also attempted to grow our *mtlR* complementation strain in mannitol in the absence of IPTG. Surprisingly, these cells harbouring the pMtlR plasmid successfully grew overnight and were able to reach mid-log phase following back-dilution. However, subsequent Western blot analysis revealed that less *mtlR* is expressed from pMtlR in *V. cholerae* grown in mannitol medium compared to non-mannitol medium (Fig. S1c). This decrease in ectopic *mtlR* expression could allow for enough MtlA to be made such that the cells are able to grow in mannitol medium. We reason that, through overnight growth, the bacteria adapted (potentially through suppressor mutations) to grow robustly in mannitol despite harbouring the pMtlR plasmid.

In wild-type *V. cholerae*, MtlR demonstrates a highly unusual expression pattern as a repressor, as expression is highest in conditions where it is not expected to be active (mannitol medium). We have yet to ascertain a mechanistic explanation for this expression profile. One possibility could be that MtlR acts as part of a regulatory complex. That is, although MtlR levels are high in mannitol, its putative protein partner(s) may be more active in non-mannitol medium, allowing for *mtlA* repression. Although MtlR structurally displays no favourable DNA-binding domains, the regulator could be acting in a larger transcriptional complex with other proteins – such as CRP or FruR – to modulate *mtlA* expression from the *mtl* locus in bacteria [31]. These potential MtlR protein-binding partners may confer the ability to bind macromolecules such as DNA or RNA, or these MtlR protein contacts could be responsible for activation and repression of MtlR activity. Our lab is currently investigating these potential binding partners.

From a functional and evolutionary standpoint, we have evidence to believe that expressing high levels of the repressor under non-repressive conditions could be advantageous for *V. cholerae*. The fact that MtlR lingers during a media switch from mannitol to non-mannitol media could mean that *V. cholerae* rely on high levels of the repressor to be present during transitions in order to accelerate adaptation to different media (Figs 6 and S8). In other words, the high levels of MtlR in mannitol medium allow the bacteria to rapidly shut-off MtlA production when the transporter is no longer needed. Indeed, one expects that in their aquatic reservoirs, *V. cholerae* would be subject to constant fluctuations, at a microscale level, of available nutrients and signalling molecules [4]. MtlR may be calibrating MtlA levels during the transition to better suit the lower extracellular levels of mannitol.

Expression of *mtlA* from the *mtl* locus is a highly regulated biological process in bacteria. The complexity of genetic regulation along the *mtl* locus is matched by the diverse functionality of MtlA in metabolic and environmental sensing processes. Mannitol is an important carbon source throughout the *V. cholerae* lifecycle; accordingly, the ability to quickly sense, acquire and process extracellular mannitol for energy remains important. In growth conditions without mannitol, though, the energy expenditure for robust expression of an unnecessary protein must be curbed. It is likely expression of *mtlA* is never fully shut off, however. *V. cholerae* may maintain MtlA protein at low levels to act as an environmental sensor for extracellular mannitol and to trigger the formation of a protective biofilm under permitting growth conditions. This complex *mtlA* expression cycle requires an equally elaborate regulation paradigm, which currently involves CRP, MtlS and MtlR.

## Supplementary Data

1504 Supplementary File 1
