# Supplementary material for: MtlR negatively regulates mannitol utilization by Vibrio cholerae
Source: Microbiology (Reading). 2017 Oct 27;163(12):1902–11. doi: 10.1099/mic.0.000559 (PMC5845740; doi:10.1099/mic.0.000559)
Supplement: Supplementary File 1 [file mic-163-1902-s001.pdf]

MtlR negatively regulates mannitol utilization by *Vibrio cholerae*

Tanner Byer, Jessica Wang, Mark G. Zhang, Anna Blachman, Naomi Vather, Bryan Visser and Jane M. Liu*

Department of Chemistry, Pomona College, Claremont, California 91711, United States
*Corresponding Author Tel: (909) 607-8832, jane.liu@pomona.edu

Supplementary Materials

Supplementary Methods
Figure S1
Figure S2
Figure S3
Figure S4
Figure S5
Figure S6
Figure S7
Figure S8
Table S1
Table S2
References

Supplementary Methods

Plasmid and strain construction

Plasmids harboring *mtlR-His*$_6$ under the control of the P_{trc} promoter were constructed by PCR amplifying *mtlR* from *V. cholerae* N16961 Δ*tcpA*. PCR products and vector pTrc99A were integrated by Gibson Assembly to produce plasmids containing P_{trc}-*mtlR-His*$_6$ alleles. A His6–*mtlR* construct was made by splicing by overlap extension (SOE)-PCR (1). Briefly, DNA fragments of approximately 600 bp upstream and downstream of the insertion were amplified by PCR from *V. cholerae* N16961 genomic DNA, annealed together by complementary sequences in the R1 and F2 primers, and then PCR amplified with the F1 and R2 primers. The final PCR product was cloned into the multiple cloning site of pCVD442 using SphI and SacI restriction sites. The *mtlR'* (early stop codon) mutants were constructed using the pCVD::His6-*mtlR* plasmid as a starting template. A stop codon was inserted at the 9th codon after the annotated start codon using the QuikChange Site-Directed Mutagenesis Kit (Stratagene) to create pCVD::His6*mtlR'1*. Stop codons were introduced further downstream of the annotated start codon by amplifying the entire pCVD–His6*mtlR* with primers that introduced the stop codon at the desired location in the *mtlR* gene. The PCR products were phosphorylated with T4 PNK and then circularized with T4 DNA ligase. All pCVD442-derived constructs were conjugated into *V. cholerae* N16961 Δ*tcpA* from *E. coli* SM10λpir as described previously (2). After one passage in LB broth with streptomycin, sucrose-resistant colonies were selected and subsequently screened for the desired mutation by PCR and sequencing.

Northern blot analysis

Total RNA was extracted from *V. cholerae* cultured to an OD_{600} ~0.3 using acid phenol: chloroform, as previously described (3). The concentration of RNA was measured using a Take-3 system (Bio-Tek) and specific mRNAs were detected using the NorthernMax-Gly Kit (Thermo Fisher Scientific). RNA (10-25 µg) was resolved on a 1.0% agarose gel via electrophoresis at 100 V for 55 min. The RNA was transferred to a 0.45 µm pore-size Hybond-N+ membrane (GE Healthcare Life Sciences), which was prehybridized for 30 min in UltraHyb Oligo solution at 68 °C (Thermo Fisher Scientific). Hybridizations were performed using either oligonucleotide probes (~50 ng) that were 5' end labeled with an IR dye, or with RNA probes (~1 pmol) transcribed from PCR-derived templates using biotin-16-UTP and T7 RNA polymerase (Promega), according to the manufacturer's instructions. The Odyssey northern blot analysis protocol was followed for detection of the probes on the membranes using an Odyssey imager (Licor).

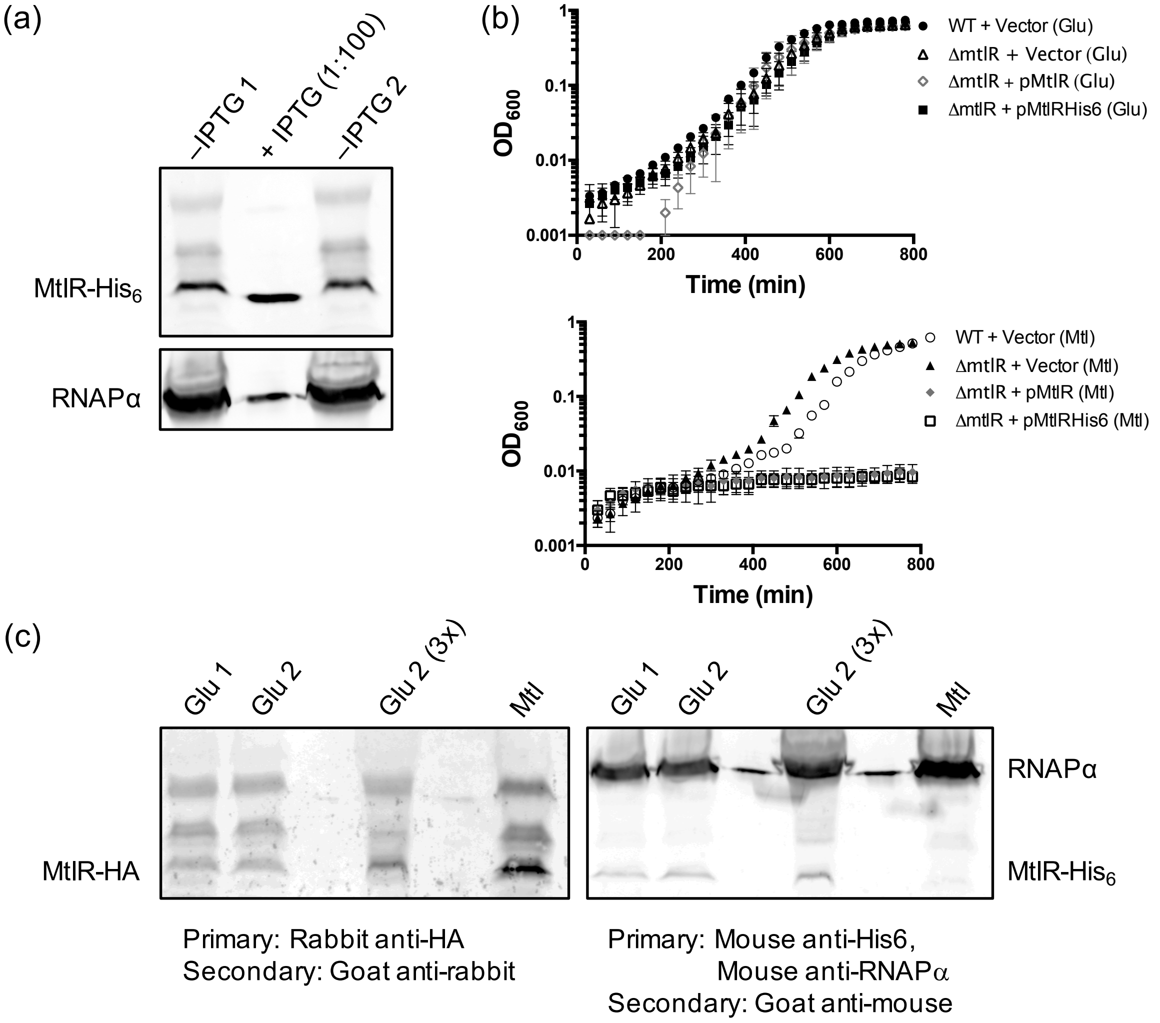

Figure S1. Ectopic expression of *mtlR* from plasmid pMtlR-His$_6$.
(a) Wild-type *V. cholerae* harboring a plasmid that expresses *mtlR* with a C-terminal hexahistidine tag from a P$_{trc}$ promoter was grown in glucose medium with or without IPTG (1 mM) to at OD$_{600}$ ~0.3. Two replicates of the –IPTG condition are shown; a 1:100 dilution of the +IPTG was used. (b) The original pMtlR plasmid used in this study and the pMtlR-His6 plasmid used to confirm ectopic expression produce similar phenotypes with regard to growth in minimal medium. The data here are the same as in Figure 1a and 1b, with the data for Δ*mtlR* pMtlR-His$_6$ added. (c) Wild type *V. cholerae mtlR*-HA harboring pMtlR-His$_6$ was back-diluted from overnight cultures and grown in minimal medium with glucose (Glu) or mannitol (Mtl) without IPTG to an OD$_{600}$ ~0.3. An equal number of cells were analyzed by western blot (for the second glucose sample, a three-times concentrated sample was also included). The blot was analyzed using dual-color detection for both MtlR-HA, encoded on the chromosome, and MtlR-His$_6$, encoded on the plasmid. RNAPα served as the loading control. As MtlR is known to dimerize, the multiple bands may represent aggregation of the protein, particularly in the presence of excess MtlR. The lack of MtlR-His$_6$ in the mannitol sample suggests that during overnight growth, the cells were able to decrease ectopic expression of *mtlR* (potentially through suppressor mutations) in order to survive in mannitol medium.

ATGATATCATCAGACGCCAAGGTCAAGATACAAAATTTTGGCCGTTTCTTATCAAACATGGTTA
TGCCGAATATTGGCGCTTTTATTGCGTGGGGCTTTATTACTGCGCTTTTTATTCCAACAGGCTG
GGTACCCAATGAGACGTTAGCCTCTTTGGTTGGGCCTATGATTACCTACTTATTGCCACTGTTG
ATCGGTTATACCGGCGGTAAACTGGCAGGTGGCGAGCGCGGTGCGGTAGTGGGTGCCATCACCA
CGATGGGCGTGATTGTCGGTACCGACATTCCCATGTTCATGGGCGCGATGATCGTCGGTCCTAT
GGGTGGCTGGGCAATTAAGGCGTTCGATAAAAAAATCGATGGTAAAGTGCGCAGTGGCTTTGAA
ATGTTGGTCAACAATTTTTCTGCCGGCATTATCGGTATGCTATGTGCCATCATCGCTTTCTTCC
TGATCGGCCCATTTGTGAAAGTGCTGTCAGGCGCATTAGCGGCTGGAGTTAACTTCCTCGTGAC
CGCTCACCTACTCCCTCTGACGTCCATTTTTGTTGAGCCAGCCAAAATTTTGTTCCTCAACAAC
GCGATTAACCACGGTATTTTCTCACCGCTGGGCATTCAGCAAGCCAGTGAAACAGGTCAATCTA
TTTTCTTCTTAATTGAGGCCAACCCAGGTCCTGGCCTTGGTATTTTGCTGGCGTACATGGTGTT
TGGTAAGGGGACAGCTCGCCAAACCGCGGGTGGCGCGACCATCATTCACTTCTTTGGTGGTATT
CACGAAATCTATTTCCCTTACATCCTAATGAATCCACGCCTGATTTTGGCGGCGATTGCGGGCG
GTATGACCGGTGTGTTTACGCTCACCGTATTTAATGCTGGTCTGGTATCTCCAGCATCTCCGGG
CTCTATTTTCGCGGTACTGCTGATGACTAATAAGGGCTCAATCTTAGGTGTAGTGTGTTCCATC
TTTGCAGCGGCAGCGGTTTCTTTTACCGTCGCAGCGCTACTGATGAAAGCACAAACTTCAACCG
AGCAAGATGGCGATAAAGACGCGTTGGTGAAAGCCACCTCGATAATGCAAGAGATGAAAGCGGG
TTCAAAAGGTCAAGCGGCACCGACGGCGACCCAAAGCAAAAAAATCGACATGGCGAATGTACAA
AGCATTATTGTCGCTTGTGATGCGGGTATGGGCTCTAGTGCCATGGGTGCCAGCATGCTACGTA
AAAAAATTCAGGAAGTCGGTTTGCCCGTTACGGTAACGAATATGGCCATCAACTCACTCCCTGC
TCATGTGGATATGGTGATCACCCACCAAGATTTGACGGACCGTGCACGTCAACACGCGCCGAAT
GCCGAGCATATTTCACTCAATAATTTCCTAGATAGCGCGTTGTACAACCAGTTAGTGACTCAAC
TTCTGGCCGCAAAGCGCCAAGCGGCCAATGATAGTCAGCTGATCAAGCCTTCGATTTTGGCCGC
TAACGATGACCGCTATGAAGTACAGCAGCCAAGCGTATTCCAACTGCAAAAGGAGAACATTCAC
CTTGGCCTGAACGCCAAAAATAAAGAGGAGGCGATTCGCTTCGCGGGTAATAAACTAGTTGAAC
TGGGTTACGTCCACCCAGAGTACGTCGATGCCATGTTCGAGCGTGAAAAGCTGGTTTCAACCTA
CCTTGGTGAGTCCATTGCCGTACCGCACGGTACTGTGGATGCCAAAGATCGCGTGATTAAAACC
GGCATTGTGATTTGCCAATACCCACAAGGCGTCGCTTTTAGTGAAGACAGCGGCGATGTCGCCA
AACTCGTGATTGGTATCGCTGCCAAGAATGATGAACATATTCAAGTCATTACCACCATTACCAA
TGCCCTCGATGACCCAAATGCTATCGATAAACTCACCTCCACCAAGGATGTGAGTGATGTATTG
AGCATTTTGGCCACCAGCCAAGCGGCATAA

Figure S2. Sequence of *mtlA* coding region. The original riboprobe used to analyze *mtlA* mRNA levels is complementary to the region of *mtlA* in orange text (4). The new riboprobe used to analyze *mtlA* in this work is complementary to the region of *mtlA* in blue text. The primers used to create the templates for the riboprobes bind to the sequences highlighted in yellow.

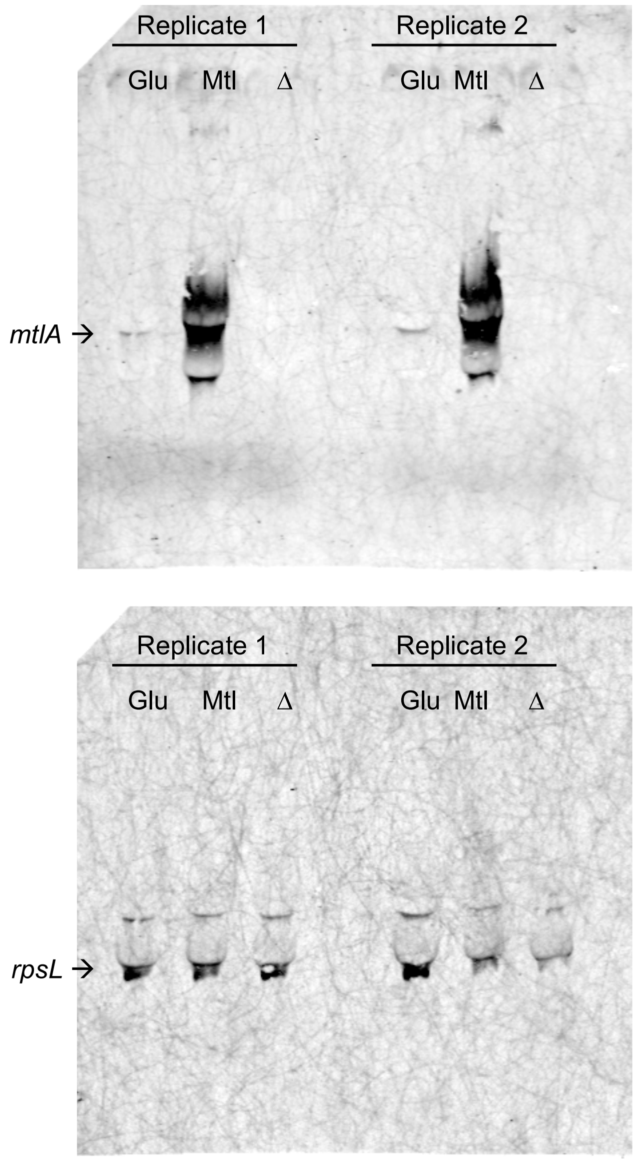

Figure S3. Analysis of *mtlA* mRNA levels in *V. cholerae*. Levels of *mtlA* mRNA in wild-type bacteria (N16961 Δ*tcpA mtlA*-FLAG) or an isogenic Δ*mtlA* strain were examined by northern blot analysis. The wild-type cells were grown in either glucose (Glu) or mannitol (Mtl) medium, and the Δ*mtlA* strain (Δ) was grown in glucose medium as the mutant is unable to grow in mannitol medium. Total RNA was collected at an OD_{600} ~ 0.3 and two biological replicates for each experimental condition are shown. The top panel is an image of the membrane probed with a riboprobe specific to *mtlA* mRNA. The bottom panel is an image of the same membrane probed with an oligonucleotide probe specific to *rpsL*, which served as a control. Multiple bands may represent mRNA processing; the most prominent band is highlighted as the transcript of interest.

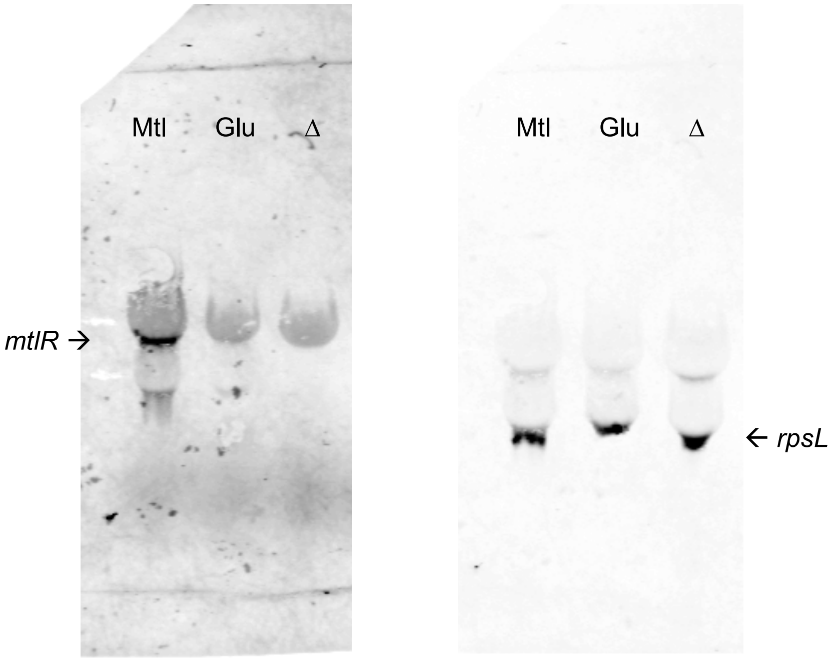

Figure S4. Analysis of *mtlR* mRNA levels in *V. cholerae*. Levels of *mtlR* mRNA in wild-type bacteria (N16961 Δ*tcpA mtlA*-FLAG) or an isogenic Δ*mtlR* strain were examined by Northern blot analysis. The wild-type cells were grown in either glucose (Glu) or mannitol (Mtl) medium, and the Δ*mtlR* strain (Δ) was grown in mannitol medium. Total RNA was collected at an OD_{600} ~ 0.3. The left panel is an image of the membrane probed with a riboprobe specific to *mtlR* mRNA. The right panel is an image of the same membrane probed with an oligonucleotide probe specific to *rpsL*, which served as a control. Multiple bands may represent mRNA processing; the most prominent band is highlighted as the transcript of interest.

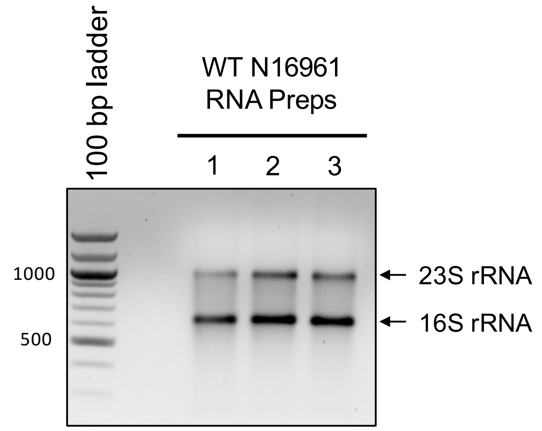

Figure S5. Total RNA (1 µg) was separated on a 1% agarose gel and stained with ethidium bromide for visualization. The three RNA samples represent biological replicates of *V. cholerae* N16961 cells cultured to an OD_{600} of 0.3 at 37 °C in M9 minimal medium with mannitol (0.4%). RNA sample 2 was used for the RT-PCR analysis in the main manuscript.

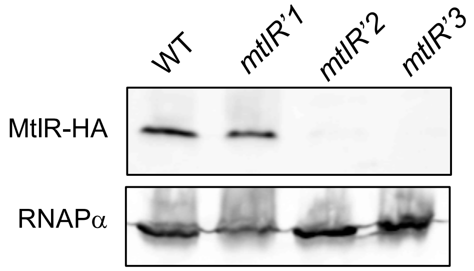

Figure S6. In our initial efforts to detect MtlR protein levels using an N-terminal epitope-tag introduced to the chromosomal copy of *mtlR*, we never observed any N-terminal-tagged MtlR in our cell lysates. Upon moving to a C-terminal HA- or FLAG-tag, however, we were able to observe MtlR protein. These results led us to question whether the N-terminus of MtlR was correctly annotated. To investigate, we introduced a stop codon nine codons after each of the three different ATG codons in the first half of the annotated *mtlR* coding sequence (creating *mtlR'1*, *mtlR'2* and *mtlR'3*, respectively). All strains include an HA-epitope at the C-terminus of *mtlR*, directly preceding the stop codon. MtlR-HA protein levels were analyzed by western blot from bacteria cultured in M9 minimal medium with mannitol (0.4%), at 37 °C to an OD_{600} of 0.3. WT, wild type *V. cholerae*. Cell lysates from an equal number of cells were analyzed using anti-HA and anti-RNAPα antibodies. RNAPα serves as a loading control. Data are representative of three independent trials.

```
  1 ATG TCG AGA GCT GTC ACT CGC TCT CGA CTT AAC TCA ACA ACA GAT
    M   S   R   A   V   T   R   S   R   L   N   S   T   T   D

 46 CAG TTT ATG GCA GAA AAA ATT AAC GAA TCC GAC ATT CTG GAG CGC
    Q   F   M   A   E   K   I   N   E   S   D   I   L   E   R

 91 TTG AAT CAG ACC CAC ACG GTA CGA GGA TTC TTC ATT ACA ACG GTT
    L   N   Q   T   H   T   V   R   G   F   F   I   T   T   V

136 GAT GTG CTA ACC GAA GCC ATT GAT GCA CTG ATG CAA CGT ATT TTC
    D   V   L   T   E   A   I   D   A   L   M   Q   R   I   F

181 CGT AAA GAC AAT TTC GCG GTG AAA TCC GTA GTT GAA CCA CTC TTG
    R   K   D   N   F   A   V   K   S   V   V   E   P   L   L

226 CAC GAT ACA GGA CCG CTG GGT GAC CTA ACG GTT CGC CTG AAA CTT
    H   D   T   G   P   L   G   D   L   T   V   R   L   K   L

271 TTA TTT GGT TTG GGC GTG ATC CCA GAT GAG GTT TTC CAC GAT ATT
    L   F   G   L   G   V   I   P   D   E   V   F   H   D   I

316 GAA CAC TTA ATC AAA CTG CGC AAT CAG CTC AAT CAT GAT GCA ACC
    E   H   L   I   K   L   R   N   Q   L   N   H   D   A   T

361 GAG TAC CAA TTC ACC GAC CCG CAA ATT CTC GCG CCA ATC AAA GCA
    E   Y   Q   F   T   D   P   Q   I   L   A   P   I   K   A

406 CTC AAT CTG GTC AAA AAA ATG GGC ATG TTG CAT TTA AAC GTC GTG
    L   N   L   V   K   K   M   G   M   L   H   L   N   V   V

451 GAG CCA GAC GAC GAT ATT GAC CTC AGC TTT TAC CAC CTG CAA TTG
    E   P   D   D   D   I   D   L   S   F   Y   H   L   Q   L

496 CAA CGC CAA CAG CAA GTG ATC AAA TCT GGT CTC TCT CTG GCC ATC
    Q   R   Q   Q   Q   V   I   K   S   G   L   S   L   A   I

541 ATT CAA ATT TGT AAT GCA CTC AAC AAA GAC AGC CCG TTT TAA
    I   Q   I   C   N   A   L   N   K   D   S   P   F   *
```

Figure S7. The annotated coding sequence of *mtlR* (obtained from KEGG), starting with the annotated start codon. All other in-frame ATG codons are highlighted in blue; the stop codon is in red. The second stop codon (**ATG** GCA GAA….) was identified as the true start codon for *mtlR*.

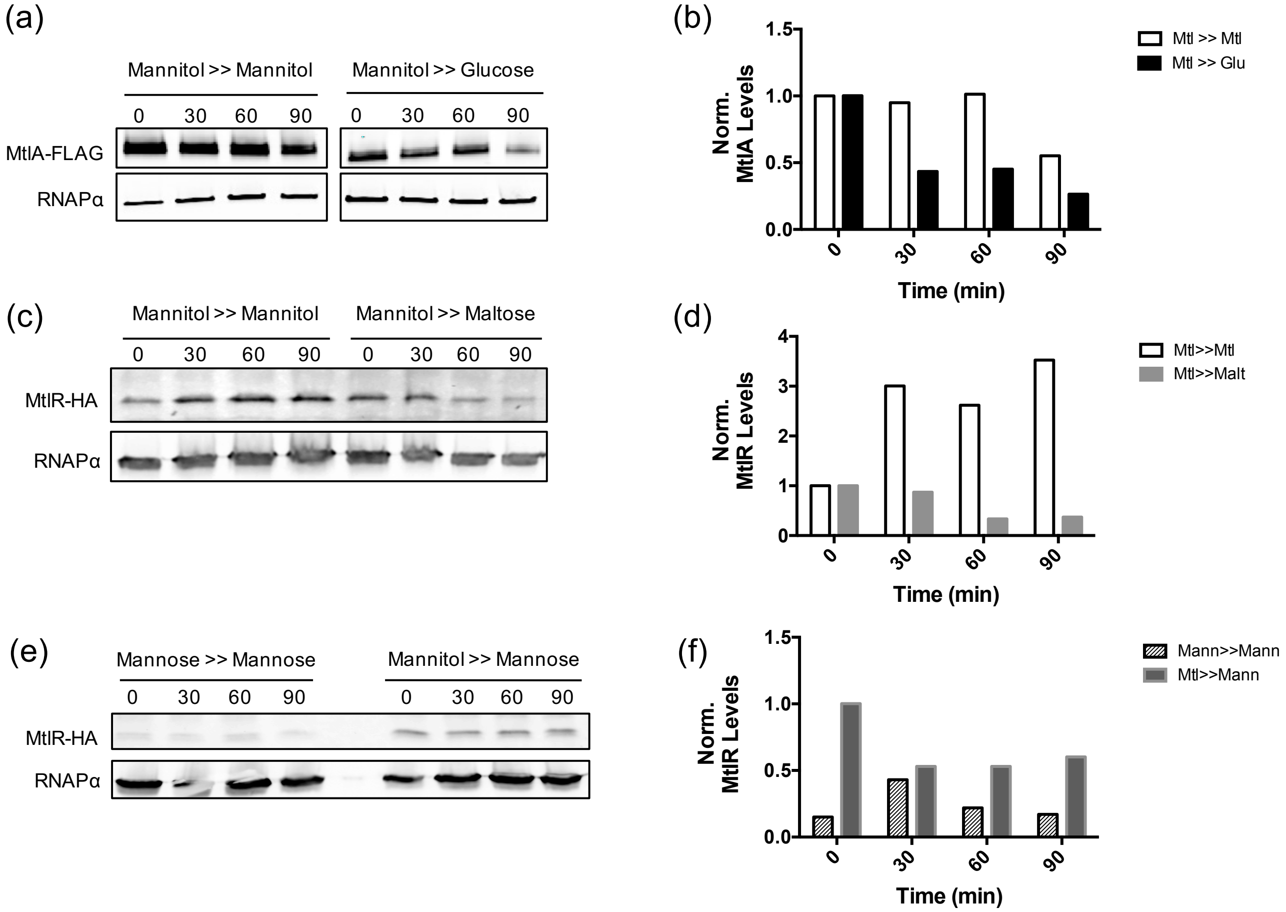

Figure S8. MtlA and MtlR protein levels decrease upon shift from mannitol to non-mannitol medium. For each western blot, whole cell lysates from an equal number of cells were analyzed with anti-FLAG and anti-RNAPα antibodies. RNAPα served as the loading control. Data are representative of at least two experiments.

(a) MtlA-FLAG protein levels in wild type *V. cholerae* cultured at 37 °C in M9 minimal medium with 0.4% mannitol. At an OD_{600} of 0.3, cells were re-suspended in an equal volume of M9 minimal media with 0.4% mannitol or glucose and harvested at 0, 30, 60, and 90 minutes relative to the media switch.

(b) Quantification of MtlA-FLAG from western blot analysis in (a), normalizing to RNAPα levels and then to MtlA-FLAG levels at time point 0.

(c) MtlR-HA protein levels in wild type *V. cholerae* shifted from mannitol to mannitol or maltose medium, as in (a).

(d) Quantification of MtlR-HA from western blot analysis in (c), normalizing to RNAPα levels and then to MtlR-HA levels at time point 0.

(e) MtlR-HA protein levels in wild type *V. cholerae* shifted from mannose or mannitol medium to mannose medium, as in (a).

(f) Quantification of MtlR-HA from western blot analysis in (e), normalizing to RNAPα levels and then to MtlR-HA in mannitol medium at time point 0.

Table S1. Bacterial strains and plasmids used in this study

Strain or plasmid	Description	Ref or Source
V. cholerae		
JL2	N16961 Δ*tcpA mtlA–flag* Sm^R	(4)
JL55	N16961 Δ*tcpA mtlA–flag* Δ*mtlR* Sm^R	(4)
JL116	N16961 Δ*tcpA* Δ*mtlA* Sm^R	(3)
JL154	N16961 Δ*tcpA mtlA–flag mtlR–HA* Sm^R	This study
JL190	N16961 Δ*tcpA mtlA–flag* Δ*mtlR* pTrc99A Sm^RCb^R	This study
JL191	N16961 Δt*cpA mtlA–flag* Δ*mtlR* pTrc99A::*mtlR* Sm^RCb^R	This study
JL198	N16961 Δ*tcpA mtlA–flag* pTrc99A Sm^RCb^R	This study
JL199	N16961 Δ*tcpA mtlA–flag* pTrc99::*mtlR* Sm^RCb^R	This study
JL260	N16961 Δ*tcpA mtlA–flag mtlR'1–HA* Sm^R	This study
JL263	N16961 Δ*tcpA mtlA–flag mtlR'2–HA* Sm^R	This study
JL264	N16961 Δ*tcpA mtlA–flag mtlR'3–HA* Sm^R	This study
JL346	C6706 Δ*tcpA* pTrc99A Sm^RCb^R	This study
JL347	C6706 Δ*tcpA* pTrc99A::*mtlR* Sm^RCb^R	This study
JL418	N16961 Δ*tcpA mtlA–flag* Δ*mtlR* pTrc99A::*mtlRHis6* Sm^RCb^R	This study
JL419	N16961 Δ*tcpA mtlA–flag* mtlR–HA pTrc99A::*mtlRHis6* Sm^RCb^R	This study
JL420	N16961 Δ*tcpA mtlA–flag* pTrc99A::*mtlRHis6* Sm^RCb^R	This study
E. coli		
DH5α	F– Φ80*lac*ZΔM15 Δ(*lac*ZYA-*arg*F) U169 *rec*A1 *end*A1 *hsd*R17 (rK–, mK+) *pho*A *sup*E44 λ– *thi*-1 *gyr*A96 *rel*A1	Laboratory strain
TOP10	F-*mcrA* Δ(*mrr-hsd*RMS-*mcr*BC) Φ80*lac*ZΔM15 Δ*lac*X74 *rec*A1 *ara*D139 Δ(*araleu*)7697 *gal*U *gal*K *rps*L (StrR) *end*A1 *nup*G	Invitrogen
DH5αλpir	F^-Δ(*lacZYA-argF*)*U169 recA1 end A1 hsdR17 supE44 thi-1 gyrA96 relA1 λ::pir*	Laboratory strain
SM10λpir	*thi recA thr leu tonA lacY supE* RP4-2-Tc::*Mu λ*::*pir*	Laboratory strain
Plasmids		
pCVD442	*oriR6K mobRP4 sacB*, Cb^R	(1)
pTrc99A	pBR322 origin, *lacI*, P_{trc} promoter, Cb^R	Laboratory plasmid
pTrc99A::*mtlR*	Entire coding sequence of *mtlR*, including 123 bp upstream of correct ATG start codon cloned after P_{trc} promoter of pTrc99A vector, Cb^R	This study
pTrc99A::*mtlRHis6*	Identical to pTrc99A::*mtlR* except the addition of a hexahistidine encoding sequence directly preceding the stop codon of *mtlR*, Cb^R	This study

Sm^R, streptomycin resistance; Cb^R, carbenicillin resistance

Table S2. Primers and probes used in this study

Name	Description	Sequence (5' → 3')
Primers		
040608A	Forward primer to insert *mtlR* into pTrc99A	G*GA GCT C*GC TCT CTA CCA ACA GAT GGA TTG
040608B	Reverse primer to insert *mtlR* into pTrc99A	G*TC TAG A*TT AAA ACG GGC TGT CTT TGT TGA GTG
LIU36N	F1 for His6-*mtlR*	GGC *GCA TGC* GTC ACT GTG TGA CGG CTT ATC TG
LIU37N	R1 for His6-*mtlR*	GCT CTC GAA TGA TGA TGA TGA TGA TGC ATT TTA AGA CTA CCG ATA ACC GCA TTT TTT C
LIU38N	F2 for His6-*mtlR*	GTA GTC TTA AAA TGC ATC ATC ATC ATC ATC ATT CGA GAG CTG TCA CTC GCT CTC
LIU39N	R2 for His6-*mtlR*	GGC *GAG CTC* AAA CGG GCT GTC TTT GTT GAG TGC
LIU40N	F0 for His6-*mtlR*	GAA GCT CTC TAC CAA CAG ATG GAT TG
LIU41N	R0 for His6-*mtlR*	CTG CCA TAA ACT GAT CTG TTG TTG AG
LIU70	F0' for *mtlR*	ATC GCA TTG TGC CAC CGT TGC
LIU71	R0' for *mtlR*	GCT TCA GGC TAA CAC GAT ACG C
mtlR_HA_Up	gBlock for *mtlR-HA*	G*GCATGC*ATTCTTCATTACAACGGTTGATGTGCTAACCGAAGCCATTGATGCACTGATGCAACGTATTTTCCGTAAAGACAATTTCGCGGTGAAATCCGTAGTTGAACCACTCTTGCACGATACAGGACCGCTGGGTGACCTAACGGTTCGCCTGAAACTTTTATTTGGTTTGGGCGTGATCCCAGATGAGGTTTTCCACGATATTGAACACTTAATCAAACTGCGCAATCAGCTCAATCATGATGCAACCGAGTACCAATTCACCGACCCGCAAATTCTCGCGCCAATCAAAGCACTCAATCTGGTCAAAAAAATGGGCATGTTGCATTTAAACGTCGTGGAGCCAGACGACGATATTGACCTCAGCTTTTACCACCTGCAATTGCAACGCCAACAGCAAGTGATCAAATCTGGTCTCTCTCTGGCCATCATTCAAATTTGTAATGCACTCAACAAAGACAGCCCGTTTGCTAGCTACCCATACGACGTCCCAGACTAC
mtlR_HA_Down	gBlock for *mtlR-HA*	GCTAGCTACCCATACGACGTCCCAGACTACGCTTAACGTTTAGGTGCACGCCGATTTCGCGTGTTCTCCGCTCAACTTCCACATCCATTTTTCATCTTGTTTGCGTATCGTGTTAGCCTGAAGCCTTTCTGATGACAAACGAGTGAAGCATGAAAATCGCTATGATTGGTCTGGGAGATATTGCCCAGAAAGCTTATCTTCCCGTATTGGCTCAATGGCCAGATATTGAATTGGTGCTCTGCACCCGAAATCCGAAAGTATTGGGGACTCTAGCGACGCGTTACCGAGTCAGCGCGACGTGCACCGACTACCGTGATGTGTTGCAGTATGGTGTGGATGCAGTGATGATCCATGCGGCGACCGAT

Table S2 cont.

		GTACACAGCACACTTGCGGCGTTTTTTC TGCATCTAGGGATCCCAACTTTTGTCGA TAAACCACTCGCCGCGAGCGCGCAAGA GTGTGAAAACTTGTATGAGTTAGCGGAG AAACATCACCAACCAC*GAGCTC*GG
LIU80	F0 for *mtlR-HA*	GCA ACG CCA ACA GCA AGT GAT C
LIU81	R0 for *mtlR-HA*	GAT GTG GAA GTT GAG CGG AGA AC
LIU90	F1 for *mtlR-HA*	G*GC ATG C*AT TCT TCA TTA CAA CGG TTG
LIU92	R2 for *mtlR-HA*	CC*G AGC T*CG TGG TTG GTG ATG
LIU161	Quik-change primer for *mtlR'1* (sense)	GCT GTC ACT CGC TCT CGA TAA CTT AAC TCA ACA ACA GAT C
LIU162	Quik-change primer for *mtlR'1* (antisense)	GAT CTG TTG TTG AGT TAA GTT ATC GAG AGC GAG TGA CAG C
LIU246	Forward primer to make pCVD::*mtlR'2*	TAA ATT CTG GAG CGC TTG AAT CAG ACC
LIU247	Reverse primer to make pCVD::*mtlR'2*	GTC GGA TTC GTT AAT TTT TTC TGC CAT AAA C
LIU248	Forward primer to make pCVD::*mtlR'3*	TAA TTC GCG GTG AAA TCC GTA GTT GAA C
LIU249	Reverse primer to make pCVD::*mtlR'3*	ATT GTC TTT ACG GAA AAT ACG TTG CAT CAG
LIU394	Forward primer for RT-PCR *mtlA*	ACT GCG CTT TTT ATT CCA ACA GGC
LIU395	Reverse primer for RT-PCR *mtlA*	AAG GCC AGG ACC TGG GTT G
LIU396	Forward primer for RT-PCR *mtlD*	GCT GGC AAC ATT GGC CGT G
LIU397	Reverse primer for RT-PCR *mtlD*	GGT TTT CTC CAT TCC CTC AAT CTG
LIU398	Forward primer for RT-PCR *mtlD*	GAA AAA ATT AAC GAA TCC GAC ATT CTG G
LIU399	Reverse primer for RT-PCR *mtlD*	CTG TCT TTG TTG AGT GCA TTA CAA ATT TG
LIU423	Reverse primer for vector to make pMtlR-His6	GAG CTC GAA TTC CAT GGT CTG TTT C
LIU424	Forward primer for vector to make pMtlR-His6	TTT CAT CAT CAC CAT CAC CAT TAA TCT AGA GTC GAC CTG CAG GC
LIU425	Forward primer for insert to make pMtlR-His6	AAC AGA CCA TGG AAT TCG AGC TC
LIU426	Reverse primer for insert to make pMtlR-His6	TCT AGA TTA ATG GTG ATG GTG ATG ATG AAA CGG GCT GTC TTT GTT GAG TG
T7 mtlA probe forward	Forward primer to make *mtlA* riboprobe template	GGA TCC TAA TAC GAC TCA CTA TAG GGT CCT TGG TGG AGG TGA GTT TAT C
mtlA probe reverse	Reverse primer to make *mtlA* riboprobe template	GCC AAA GAT CGC GTG ATT AAA ACC
T7 mtlR probe forward	Forward primer to make *mtlR* riboprobe template	GGATCCTAATACGACTCACTATAGGG GTC AAT ATC GTC GTC TGG CTC CAC
mtlR probe reverse	Reverse primer to make *mtlR* riboprobe template	AGG ACC GCT GGG TGA CCT AAC

Table S2 cont.

Oligonucleotides and qPCR probes		
800-rpsL	Probe for *rpsL* RNA	IRD800-CTT GCT TAG CAC GTG GCT TAC GAA CC
mtlA forward	Forward qPCR primer for *mtlA*	TCC CCC GTT GGA TGT TCC G
mtlA reverse	Reverse qPCR primer for *mtlA*	CCG TTG GTG ATT CCA TTC G
mtlR forward	Forward qPCR primer for *mtlR*	TGA TCC CAG ATG AGG TTT TCC
mtlR reverse	Reverse qPCR primer for *mtlR*	GAT TGA GTG CTT TGA TTG GCG
4.5S forward	Forward qPCR primer for 4.5S	CTG GTC CTC CCG CAA CAC
4.5S reverse	Reverse qPCR primer for 4.5S	GAG ACC CCA GCC ACA TC

Italicized: restriction site
Underlined: epitope tag sequence or T7 promoter sequence

References

1. Donnenberg MS, Kaper JB. Construction of an *eae* deletion mutant of enteropathogenic *Escherichia coli* by using a positive-selection suicide vector. *Infect Immun.* 1991;59(12):4310–7.

2. Lee SH, Angelichio MJ, Mekalanos JJ, Camilli A. Nucleotide sequence and spatiotemporal expression of the *Vibrio cholerae vieSAB* genes during infection. *J Bacteriol*. 1998;180(9):2298–305.

3. Chang H, Replogle JM, Vather N, Tsao-Wu M, Mistry R, Liu JM. A cis-regulatory antisense RNA represses translation in *Vibrio cholerae* through extensive complementarity and proximity to the target locus. *RNA Biol*. 2015;12(2):136–48.

4. Mustachio LML, Aksit SS, Mistry RHR, Scheffler RR, Yamada AA, Liu JMJ. The *Vibrio cholerae* mannitol transporter is regulated posttranscriptionally by the MtlS small regulatory RNA. *J Bacteriol*. 2012;194(3):598–606.
